# Factors associated with smoking cessation and relapse in the Japanese smoking cessation treatment program: A prospective cohort study based on financial support in Suita City, Japan

**DOI:** 10.18332/tid/112154

**Published:** 2019-10-02

**Authors:** Meng Li, Reiko Okamoto, Fumie Shirai

**Affiliations:** 1 Public Health Nursing Laboratory, Division of Health Sciences, Graduate School of Medicine, Osaka University, Suita, Japan; 2 Community Health-Care System Science Laboratory, Division of Health Sciences, Graduate School of Medicine, Osaka University, Suita, Japan

**Keywords:** smoking cessation, Japan, treatment adherence, financial support

## Abstract

**INTRODUCTION:**

The purpose of this study is to clarify the effect of providing financial support and factors associated with smoking cessation and relapse in the Japanese smoking cessation treatment (SCT) program based on financial support.

**METHODS:**

A prospective cohort study was conducted at the smoking cessation outpatients of hospitals or clinics in Suita City, Japan from May 2017 to September 2018. In all, 153 participants were recruited and received standardized treatment based on the SCT program. Participants were required to answer four questionnaires and register for the financial support program. Chi-squared test, Fisher’s exact test, non-paired t-test and log-binomial regression analysis were used to analyze the data.

**RESULTS:**

Of the 153 participants, 140 participants completed a 12-week treatment and the completion rate was 91.5%. There were no factors significantly associated with smoking cessation and relapse (p<0.05). However, male, cigarettes smoked per day, having present diseases, having previous abstinence, living with family, cohabitation with smokers, desire to smoke at the start of treatment, self-efficacy at the start of treatment, desire to smoke at 4 weeks and self-efficacy at 4 weeks showed statistically significant odds ratio for success of smoking cessation. Similarly at 12 weeks, male, age at smoking initiation, cigarettes smoked per day, having previous abstinence, living with family, cohabitation with smokers, desire to smoke, self-efficacy and depression disorders showed statistically significant odds ratio for smoking relapse. In addition, the rate of abstainers using varenicline was 68.60%, which was higher than abstainers using nicotine patch (55.60%) and the relapse rate of participants using nicotine patch was 100.00%, significantly higher than for relapsers using varenicline (45.80%).

**CONCLUSIONS:**

Further study is expected to clarify the effect of providing financial support and the factors associated with smoking cessation and relapse in the SCT program based on financial support.

## INTRODUCTION

Tobacco use is a significant public health concern worldwide and one of the major causes of death and disability in both developed and developing countries. According to the 2017 World Health Organization (WHO) report^1^, tobacco use is currently responsible for the death of about 7 million people worldwide each year, including 600 thousand deaths due to passive smoking. To reduce health problems due to smoking or passive smoking, WHO has enacted the Framework Convention on Tobacco Control (FCTC) in the World Health Assembly, 21 May 2003, and the convention gives specific recommendations for different tobacco control strategies such as developing comprehensive smoking cessation guidelines and introducing warning labels on cigarette packages^2^. In these strategies, one approach to promote tobacco control is to take effective measures to promote smoking cessation and offer an adequate treatment program for tobacco dependence.

In Japan, tobacco use has become the leading preventable cause of mortality and morbidity, resulting in about 129 thousand deaths^3^. In light of this troubling fact, the Japanese medical insurance system has covered the smoking cessation treatment (SCT) program for nicotine dependence since 2006. Until March 2018, more than 16400 facilities have established smoking cessation outpatients or clinics, and the treatment population has been expanded to young people under 35 years of age, regardless of the Brinkman Index. With the development of smoking cessation treatment in Japan, a large number of studies have investigated factors associated with smoking cessation and relapse in the SCT program. In addition, to improve the effects of treatment, Arakawa Ward, Tokyo was first to provide financial support for the SCT program since 2007, and thereafter a similar support program has been extended to other wards and cities. However, the effects of providing financial support and factors associated with smoking cessation and relapse in the SCT program based on financial support have not been reported. Therefore, the purpose of this study is to clarify the effects of providing financial support and factors associated with smoking cessation and relapse in the SCT program based on financial support.

## METHODS

### Design

A prospective cohort study was conducted at the smoking cessation outpatients of several hospitals or smoking cessation clinics in Suita City, Japan from May 2017 to September 2018. In all, 153 participants were recruited and received treatment based on the 6th standardized guideline^4^ of the SCT program. Meanwhile, participants were required to answer four questionnaires at the start of treatment, at 4 weeks, at 12 weeks, and at 1 year, and register for the financial support program (cost: 10000 yen, about US$92.87) within one month before or after the start of treatment. In order to earn the financial support after the treatment, three conditions must be met, as follows:

1. Applicants must complete a 12-week treatment program.
2. Applicants are limited to 100 people if over 100 people complete this program, meanwhile over 100 people applied for the financial support.
3. Applicants must submit relevant materials (financial support application form, receipt, medical expense statement, etc.) within about one month after the treatment.

The applications for financial support and the responses of four questionnaires were completed at the reception counter of the Health Center of Suita City or by mail. This study was approved by the Ethical Committee of Observation Research at the Osaka University Hospital (Approval No: 18362), and the division of roles was implemented based on a joint research contract between Suita City and Osaka University.

### The Japanese SCT program

The Japanese SCT program has been described in previous studies^5,6^. Briefly, the SCT program consists of a total of five sessions: first session and then follow-up sessions at 2, 4, 8 and 12 weeks after the first session. At each session, the current smoking behavior is confirmed by exhaled carbon monoxide (CO) concentration and the smoker’s self-reported smoking status. Meanwhile, patients receive medication treatment consisting of varenicline (standard use: 12 weeks) or nicotine patches (standard use: 8 weeks), and behavioral counselling from physicians and nurses.

### Data collection

Data collection involves four questionnaires at the start of treatment, at 4 weeks, at 12 weeks, and at 1 year. The four questionnaires were developed based on previous studies^5,6^ and specialists’ opinions from Osaka University and the Health Center of Suita City. The first questionnaire at the start of treatment includes information on age, gender, age at smoking initiation, cigarettes smoked per day, previous abstinence, self-efficacy, cohabiter, cohabitation with smokers, passive smoking, having present diseases, strength of desire to smoke, and a CES-D score (Center for Epidemiologic Studies Depression Scale). The second questionnaire at 4 weeks includes information on smoking status, self-efficacy, strength of desire to smoke, satisfaction with smoking cessation treatment, and received instructions during the period of SCT. The third questionnaire at 12 weeks includes information on smoking status at 8 weeks and 12 weeks, self-efficacy, strength of desire to smoke, satisfaction with smoking cessation treatment, received instructions during the period of SCT, CES-D score, thoughts and feelings for financial support (free description). The fourth questionnaire at 1 year includes information on smoking status at 1 year, self-efficacy, strength of desire to smoke, received instructions during the period of SCT, CES-D score, thoughts and feelings for challenge to quit smoking (free description).

### Strength of desire to smoke

Strength of desire to smoke (craving) is evaluated by grades (0: none; 1: slight; 2: moderate; 3: strong) based on the combination of two questions: ‘How many times did you want to smoke in the past two weeks?’ (with responses, 0: Not at all; 1: Several times a week; 2: Several times a day; 3: Many times a day), and ‘How strongly did you want to smoke in the past two weeks?’ (with responses, 0: Not at all; 1: Several times a week; 2: Several times a day; 3: Many times a day)^7^.

### CES-D scale

The CES-D scale is designed to measure depressive symptomatology in the general population, including 20 short self-report items. The component mainly consists of depressed mood, feelings of guilt and worthlessness, feelings of helplessness and hopelessness, psychomotor retardation, loss of appetite, and sleep disturbance. The responses of each item are weighted by frequency of occurrence of the symptom scoring from zero to three during the past week, and the possible range of CES-D scored from zero to sixty. The higher scores indicate more symptoms and over 16 points are diagnosed as being in a mood disorder group^7^.

### Self-efficacy scale

Self-efficacy in the smoking cessation treatment is defined as the confidence a person had in his/her ability to quit smoking and prevent relapse after a quit attempt. In the past, various questionnaires have been used to measure self-efficacy with regard to smoking cessation. A recent meta-analysis study reported that the effect size of self-efficacy with single-item measures was stronger than multi-item measures. Therefore, the self-efficacy scale with a single-item 0–100 rating is used to evaluated participants’ confidence for smoking cessation in this study. The self-efficacy to quit smoking is asked in four questionnaires: ‘How much, as a per cent, confidence do you have to quit smoking?’, with 0% corresponding to ‘I don’t have confidence at all to quit smoking’ and 100% corresponding to ‘I absolutely have confidence to quit smoking’^8,9^.

### Definition of smoking status

In this study, success of smoking cessation (abstainer) is defined as at least previous 4 weeks of smoking abstinence at the end of the SCT program (12 weeks) from the third self-reported questionnaire. Maintained cessation (non-relapser) is defined as smoking abstinence at 1 year from the fourth self-reported questionnaire. Participants who do not reply to the questionnaire or answer continuous smoking in the questionnaires are defined as failure of smoking cessation (non-abstainer) at 12 weeks and smoking relapse (relapser) at 1 year.

### Statistical analysis

Statistical analysis was divided into three stages. First, gender, age at smoking initiation, cigarettes smoked per day, previous abstinence, cohabiter, cohabitation with smokers, smokers at workplace or familiar places, having present diseases, strength of desire to smoke at the start of treatment, self-efficacy at the start of treatment, CES-D score at the start of treatment were presented by frequency distribution and percentage; while age, age at smoking initiation, cigarettes smoked per day, self-efficacy and CES-D score were also presented as mean and standard deviation (SD). Second, chi-squared test and non-paired t-test were used to examine the association between success of smoking cessation and age, gender, age at smoking initiation, cigarettes smoked per day, previous abstinence, cohabiter, cohabitation with smokers, smokers at workplace or familiar places, having present diseases, strength of desire to smoke at the start of treatment, self-efficacy at the start of treatment, CES-D score at the start of treatment, self-efficacy at 4 weeks, and strength of desire to smoke at 4 weeks. Subsequently, log-binomial regression analysis was used to examine factors associated with success of smoking cessation at 12 weeks. Similarly, chi-squared test and non-paired t-test were used to examine the association between smoking relapse and age, gender, age at smoking initiation, cigarettes smoked per day, previous abstinence, cohabiter, cohabitation with smokers, smokers at workplace or familiar places, having present diseases, strength of desire to smoke at 12 weeks, self-efficacy at 12 weeks, and CES-D score at 12 weeks. Subsequently, log-binomial regression analysis was used to examine factors associated with smoking relapse at 1 year. Finally, chi-squared test was used to examine the association between success of smoking cessation and smoking cessation medicine, and Fisher’s exact test was used to examine the association between smoking relapse and smoking cessation medicine. All statistical analyses were performed using IBM SPSS Statistics 25.0. Values with p<0.05 (two-tailed) were statistically significant.

## RESULTS

### Participant flow

**Figure 1:**
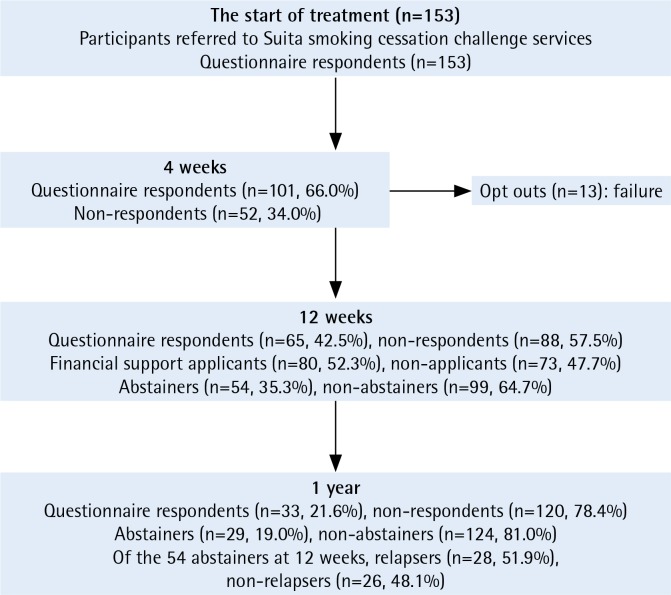
Flow of participants through study

A total of 153 participants who were screened for eligibility were referred to Suita smoking cessation challenge services from May 2017 to September 2018. At the start of treatment, all 153 participants answered the first questionnaire. At 4 weeks, 101 participants (66.0%) answered the second questionnaire. At 12 weeks, 13 participants opted out during the period of treatment, and 140 participants (91.5%) completed the 12-week treatment. At 12 weeks, 65 participants (42.5%) answered the third questionnaire, 54 participants (35.3%) were confirmed as abstainers, and 80 participants (52.3%) applied for financial support. At 1 year, 33 participants (21.6%) answered the third questionnaire, 29 participants (19.0%) were confirmed as abstainers, and of the 54 abstainers at 12 weeks, 28 abstainers (52.3%) were confirmed as relapsers (Figure 1).

### Characteristics of the participants

For the 153 participants, the mean age was 53.69 years (SD=13.056). Over 70% of the participants were male. The mean age at smoking initiation was 20.59 years (SD=3.882), and nearly 80% of the participants started smoking at 20 years of age or older. The mean number of cigarettes smoked per day was 20.57 (SD=8.108), and over 60% of the participants smoked 11–20 cigarettes per day. Nearly 40% of the participants had present diseases, and over 60% had experienced previous abstinence. Over 80% of the participants were living with family, and nearly 15% were living with smokers. About 70% of participants had moderate (32.7%) and strong (35.9%) desire to smoke at the start of treatment. The mean value of self-efficacy at the start of treatment was 61.29 (SD=8.108), and over a half of participants had low self-efficacy to quit smoking (≤60%). The mean CES-D score was 9.84 (SD=8.284), and 22.9% of participants were diagnosed as being in a mood disorder group (≥16) (Table 1).

**Table 1:** Characteristics of the participants in Suita city smoking cessation challenge services (n=153)

	*n*	*%*	*Mean*	*SD*
**Age (years)**			53.69	13.06
Gender				
Male	112	73.2		
Female	41	26.8		
**Age at smoking initiation (years)**			20.59	3.88
<20	33	21.6		
≥20	120	78.4		
**Cigarettes smoked per day**			20.57	8.11
≤10	20	13.1		
11–20	97	63.4		
21–30	26	17.0		
>30	10	6.5		
**Smoking cessation medicine**				
Not used	73	47.7		
Varenicline	70	45.8		
Nicotine patch	9	5.9		
Nicotine patch → Varenicline	1	0.7		
**Having present diseases**				
Absence	93	60.8		
Presence	60	39.2		
**Previous abstinence**				
Not experienced	54	35.3		
Experienced	99	64.7		
**Cohabiter (missing value: 2)**				
Living alone	22	14.4		
Living with family	129	84.3		
**Cohabitation with smokers (missing value: 2)**				
Absence	126	82.4		
Presence	25	16.3		
**Smokers at workplace or familiar places**				
Absence	26	17.0		
Presence	127	83.0		
**Strength of desire to smoke at the start of treatment**				
None	3	2.0		
Slight	45	29.4		
Moderate	50	32.7		
Strong	55	35.9		
**Self-efficacy at the start of treatment in %**			61.29	26.53
≤60	81	53.0		
>60 and ≤80	36	23.5		
>80 and ≤100	36	23.5		
**CES-D score at the start of treatment (missing value: 2)**			9.84	8.28
<16	116	75.8		
≥16	35	22.9		

### Factors associated with success of smoking cessation at 12 weeks

There were no factors statistically significantly associated with success of smoking cessation at 12 weeks (p<0.05). In the log-binomial regression analysis, male (OR=1.45, 95% CI: 0.67–3.14), 21–30 (OR=0.44, 95% CI: 0.12–1.69) and >30 (OR=0.80, 95% CI: 0.16–4.08) cigarettes smoked per day, having present diseases (OR=1.58, 95% CI: 0.80–3.10), having previous abstinence (OR=0.61, 95% CI: 0.31–1.22), living with family (OR=2.02, 95% CI: 0.70–5.81), cohabitation with smokers (OR=0.85, 95% CI: 0.34–2.12), slight (OR=0.33, 95% CI: 0.03–3.95), moderate (OR=0.19, 95% CI: 0.02–2.32) and strong (OR=0.29, 95% CI: 0.02–3.35) desire to smoke at the start of treatment, >60 and ≤80% (OR=0.51, 95% CI: 0.20–1.27) and >80 and ≤100% (OR=1.60, 95% CI: 0.72–3.56) self-efficacy at the start of treatment, and then moderate (OR=0.60, 95%CI: 0.15–2.45) and strong (OR=0.12, 95%CI: 0.01–1.32) desire to smoke at 4 weeks, >60 and ≤80% (OR=1.62, 95% CI: 0.50–5.20) and >80 and ≤100% (OR=2.36, 95% CI: 0.85–6.55) self-efficacy at 4 weeks showed statistically significant odds ratio for success of smoking cessation (Table 2).

**Table 2:** Factors associated with success of smoking cessation at 12 weeks in the Japanese smoking cessation treatment program

	*At start of treatment (n=153)*						
	*Nonabstainers (n=99)*	*Abstainers (n=54)*	*p*	*95% CI*	*Odds ratio*^*c*^	*95% CI*	*p*
**Age (years)^b^**							
Mean (SD)	52.83 (12.89)	55.28 (13.33)	0.27	-6.81–1.91	1.02	0.99–1.04	0.27
**Gender^a^**							
Female	29	12	0.35		1		
Male	70	42			1.45	0.67–3.14	0.35
**Age at smoking Initiation (years)**							
<20	21	12	0.89		1		
≥20	78	42			0.94	0.42–2.10	0.89
**Cigarettes smoked per day**							
≤10	13	7	0.25		1		
11–20	58	39			1.25	0.46–3.41	0.67
21–30	21	5			0.44	0.12–1.69	0.23
>30	7	3			0.80	0.16–4.08	0.78
**Having present diseases**							
Absence	64	29	0.19		1		
Presence	35	25			1.58	0.80–3.10	0.19
**Previous abstinence**							
Not experienced	31	23	0.16		1		
Experienced	68	31			0.61	0.31–1.22	0.16
**Cohabiter (missing value: 2)**							
Living alone	17	5	0.19		1		
Living with family	81	48			2.02	0.70–5.81	0.20
**Cohabitation with smokers (missing value: 2)**							
Absence	81	45	0.72		1		
Presence	17	8			0.85	0.34–2.12	0.72
**Smokers at workplace or familiar places**							
Absence	17	9	0.94		1		
Presence	82	45			1.04	0.43–2.51	0.94
**Strength of desire to smoke at the start of treatment**							
None	1	2	0.40		1		
Slight	27	18			0.33	0.03–3.95	0.38
Moderate	36	14			0.19	0.02–2.32	0.20
Strong	35	20			0.29	0.02–3.35	0.32
**Self-efficacy at the start of treatment in %**							
≤60	52	29	0.08		1		
>60 and ≤80	28	8			0.51	0.20–1.27	0.15
>80 and ≤100	19	17			1.60	0.72–3.56	0.25
**CES-D score at the start of treatment (missing value: 2)**							
<16	75	41	0.91		1		
≥16	23	12			0.95	0.43–2.11	0.91
**At 4 weeks (n=101)**							
	Non-abstainers (n=50)	Abstainers (n=51)	p	95% CI	Odds ratio^c^	95% CI	p
**Strength of desire to smoke**							
None	5	7	0.20		1		
Slight	26	32			0.88	0.25–3.10	0.84
Moderate	13	11			0.60	0.15–2.45	0.48
Strong	6	1			0.12	0.01–1.32	0.08
**Self-efficacy in %**							
≤60	14	8	0.24		1		
>60 and ≤80	13	12			1.62	0.50–5.20	0.42
>80 and ≤100	23	31			2.36	0.85–6.55	0.10

a For chi-squared test

b For non-paired t-test

c For log-binomial regression analysis: not adjusted.

### Factor associated with relapse at 1 year

 There were no factors statistically significantly associated with smoking relapse at 12 weeks (p<0.05). In the log-binomial regression analysis, male (OR=1.70, 95% CI: 0.46–6.21), ≥20 years of age at smoking initiation (OR=0.71, 95% CI: 0.20–2.61), 11–20 cigarettes (OR=0.64, 95% CI: 0.13–3.26) and 21–30 cigarettes smoked per day (OR=3.00, 95% CI: 0.21–42.62), having previous abstinence (OR=2.46, 95% CI: 0.82–7.45), living with family (OR=0.20, 95% CI: 0.02–2.11), cohabitation with smokers (OR=1.74, 95% CI: 0.37–8.18), moderate (OR=0.67, 95% CI: 0.11–3.93) desire to smoke at 12 weeks, >60 and ≤80% (OR=0.25, 95% CI: 0.02–2.95) and >80 and ≤100% (OR=0.22, 95% CI: 0.02–2.20) self-efficacy at 12 weeks and ≥16 CES-D score at 12 weeks showed statistically significant odds ratio for smoking relapse (Table 3).

**Table 3:** Factors associated with smoking relapse at 1 year in the Japanese smoking cessation treatment program

	*At start of treatment (n=54)*									
	*Nonrelapsers (n=26)*	*Relapsers (n=28)*	*p*	*95% CI*	*Odds ratio*^*c*^	*95% CI*	*p*
**Age (years)^b^**							
Mean (SD)	55.27 (13.62)	55.29 (13.31)	1.00	-7.37–7.34	1.00	0.96–1.04	1.00
**Gender^a^**							
Female	7	5	0.42		1		
Male	19	23			1.70	0.46–6.21	0.43
**Age at smoking Initiation (years)**							
<20	5	7	0.61		1		
≥20	21	21			0.71	0.20–2.61	0.61
**Cigarettes smoked per day**							
≤10	3	4	0.49		1		
11–20	21	18			0.64	0.13–3.26	0.59
21–30	1	4			3.00	0.21–42.62	0.42
>30	1	2			1.50	0.09–25.39	0.78
**Having present diseases**							
Absence	15	14	0.57		1		
Presence	11	14			1.36	0.47–3.99	0.57
**Previous abstinence**							
Not experienced	14	9	0.11		1		
Experienced	12	19			2.46	0.82–7.45	0.11
**Cohabiter (missing value: 1)**							
Living alone	1	4	0.17		1		
Living with family	25	23			0.20	0.02–2.11	0.20
**Cohabitation with smokers (missing value: 1)**							
Absence	4	5	0.81		1		
Presence	22	23			1.74	0.37–8.18	0.48
**Smokers at workplace or familiar places**							
Absence	17	9	0.94		1		
Presence	82	45			0.84	0.20–3.53	0.81
**Strength of desire to smoke**							
None	1	1	0.89		1		
Slight	8	10			1.25	0.07–23.26	0.88
Moderate	6	8			1.33	0.07–25.91	0.85
Strong	11	9			0.82	0.05–15.00	0.89
**Self-efficacy in %**							
≤60	14	15	0.61		1		
>60 and ≤80	5	3			0.56	0.11–2.79	0.48
>80 and ≤100	7	10			1.33	0.40–4.47	0.64
**CES-D score (missing value: 2)**							
<16	19	22	0.47		1		
≥16	7	5			0.62	0.17–2.27	0.47
**At 4 weeks (n=51)**							
	Non-relapsers (n=24)	Relapsers (n=27)	p	95% CI	Odds ratio^c^	95% CI	p
**Strength of desire to smoke**							
None	3	4	0.51		1		
Slight	14	18			0.96	0.19–5.03	0.97
Moderate	7	4			0.43	0.06–2.97	0.39
Strong	0	1			-	-	-
**Self-efficacy in %**							
≤60	2	6	0.40		1		
>60 and ≤80	6	6			0.33	0.05–2.37	0.27
>80 and ≤100	16	15			0.31	0.05–1.80	0.19
**At 12 weeks (n=54)**							
	Non-relapsers (n=26)	Relapsers (n=28)	p	95% CI	Odds ratio^c^	95% CI	p
**Strength of desire to smoke**							
None	8	9	0.88		1		
Slight	14	16			1.02	0.31–3.35	0.98
Moderate	4	3			0.67	0.11–3.93	0.65
Strong	0	0			-	-	-
**Self-efficacy in %**							
≤60	1	4	0.39		1		
>60 and ≤80	6	6			0.25	0.02–2.95	0.27
>80 and ≤100	19	17			0.22	0.02–2.20	0.20
**CES-D score (missing value: 1)**							
<16	23	24	0.47		1		
≥16	2	4			1.92	0.32–11.49	0.48

a For chi-squared test

b For non-paired t-test

c For log-binomial regression analysis: not adjusted.

### Smoking cessation medicine associated with smoking cessation and relapse

The association between smoking cessation medicine and smoking cessation was not statistically significant (p=0.43), while the rate of abstainers using varenicline (68.60%) was higher than abstainers using nicotine patch (55.60%) (Table 4). The association between smoking cessation medicine and relapse was statistically significant (p<0.05), and the rate of relapsers using nicotine patch was 100%, significantly higher than relapsers using varenicline (45.80%) (Table 5).

**Table 4:** Medicine associated with success of smoking cessation at 12 weeks in the Japanese smoking cessation program

	*Non-abstainers (n=26)*	*Abstainers (n=53)*	*p*
Smoking cessation medicine^a^			
Varenicline	22 (31.40%)	48 (68.60%)	0.43
Nicotine patch	4 (44.40%)	5 (55.60%)

a For chi-squared test.

**Table 5:** Medicine associated with smoking relapse at 1 year in the Japanese smoking cessation program

	*Non-relapsers (n=26)*	*Relapsers (n=27)*	*p*
Smoking cessation medicine^d^			
Varenicline	26 (54.20%)	22 (45.80%)	<0.05
Nicotine patch	0 (0.00%)	5 (100.00%)

d For Fisher’s exact test.

## DISCUSSION

To the best of our knowledge, this is the first study to discuss the effects of providing financial support and factors associated with smoking cessation and relapse, based on financial support in Japan. From many studies^10-17^ it is obvious that the completion of the SCT program improved the success of smoking cessation, while the completion rate was very low (68.0%^10^, 50.0%^11^, 36.3%^18^, 56.9%^19^, 32.4%^20^). The completion rate of the SCT program in our study was 91.5%, which is far higher than in these previous studies and in three previous surveys^15-17^ (30.0% in 2007, 35.5% in 2009, and 29.8% in 2017) reported by the Japanese Ministry of Health, Labor and Welfare. This comparison implies that providing financial support may improve the completion rate of smoking cessation programs, although this study is not a randomized controlled trial concerning the association between financial support and the completion of the SCT program.

Identification of factors associated with smoking cessation and relapse can help health care workers to improve the effectiveness of efforts to manage smoking behaviors. In regard to factors associated with smoking cessation at 12 weeks, there were no factors significantly associated with success in smoking cessation. However, we found that gender, cigarettes smoked per day, having present diseases, having previous abstinences, cohabiter, cohabitation with smokers, desire to smoke, and self-efficacy showed statistically significant odds ratio for success in smoking cessation. Similarly, our study also demonstrated that there were no factors significantly associated with smoking relapse at 1 year. However, we found that gender, age at smoking initiation, cigarettes smoked per day, having previous abstinences, cohabiter, cohabitation with smokers, desire to smoke, and self-efficacy CES-D score showed statistically significant odds ratio for smoking relapse. In addition, our study demonstrated that the rate of abstainers using varenicline was 68.60%, which was higher than abstainers using nicotine patch (55.60%) at 12 weeks, while at 1 year the relapse rate of participants using nicotine patch was 100.00%, significantly higher than relapsers using varenicline (45.80%).

In regard to gender associated with success in smoking cessation, our study demonstrated that males were more likely to quit smoking than females, and this result was consistent with previous studies^21-29^. This may be because females had more severe withdrawal symptoms, higher satisfaction levels for smoking, limited methods in releasing stress, more strongly anxious about weight gain due to quit smoking, more sales strategies targeting women and more smokers in the family compared to male smokers^21^. In general, nicotine dependence was a significant factor that prevented smoking cessation. Our study found that patients smoking 21–30 and >30 cigarettes per day found it difficult to quit smoking. This result may imply that high nicotine dependence is negatively associated with success of smoking cessation.

In our study, we found that having present diseases was positively associated with success of smoking cessation and this was consistent with previous studies^12,29^. This may be because smokers with present diseases, especially smoking-related diseases such as cardiovascular diseases, COPD, are more concerned about their health and more eager to quit smoking. However, previous studies^14,18,23,29^ demonstrated that having mental diseases was negatively associated with success of smoking cessation. This difference implies that smokers with different present diseases may have different smoking cessation behavior. In addition, we found that having previous abstinence, living alone and cohabitation with smokers were negatively associated with success of smoking cessation and this result is consistent with previous studies^5,12,14,21^.

In regard to the short-term effect (12 weeks) of varenicline treatment, previous studies demonstrated that the rate of abstainers was 43.5–84.5%^5,11,19,23,25-29,30-32^ while our study demonstrated that the rate of abstainers was 68.60%. Furthermore, with regard to the long-term effect (1 year) of varenicline treatment, previous studies demonstrated that the success rate was 46.6–68.4%^5,25,28^ while our study demonstrated that the success rate (non-relapsers) was 54.20%. In addition, a recent randomized clinical trial^33^ showed no significant differences in rates of smoking abstinence between these two treatments. However, our study demonstrated that varenicline appeared to be more effective than nicotine patch in Japan and this result is consistent with previous studies^5,11,23,25,28,32^.

To date, studies have demonstrated that self-efficacy was positively associated with success of smoking cessation among smokers who received smoking cessation intervention^6^. In Japan, four previous studies^5,6,14,23^ also demonstrated that there was a positive association between self-efficacy and the success in smoking cessation, although self-efficacy was divided into several stages based on different scores. Furthermore, our study demonstrated that participants with self-efficacy scores >80% at the first session and >60% at the second session were more likely to attain abstinence than those with low self-efficacy scores, although there was not a statistically significant association (p>0.05). These results suggest that maintaining participants’ self-efficacy at a high level is able to enhance success of smoking cessation. In addition, a review of 21 studies examining predictors of more than 6 months cessation outcome found that self-efficacy predicted relapse among both self-quitters and treated smokers^34^. Our study also demonstrated that self-efficacy scores >60% at 12 weeks were negatively associated with smoking relapse.

### Limitations

There are several limitations in this study. First, the number of participants was only 153 and this may affect the efficiency of the results. Second, we only used self-report questionnaires to assess smoking status that was not biochemically verified. Third, 60.9% of participants had a present illness in our study. Therefore, these results may not be able to be applied to the general population. Fourth, the strength scale of desire to smoke was only used in Japan and the psychometric properties of this scale do not exist.

## CONCLUSIONS

This is the first study in Japan to discuss the effects of providing financial support and factors associated with smoking cessation and relapse, based on financial support. Our results suggest that providing financial support may be able to improve the completion rate of the SCT program. Male, cigarettes smoked per day, having present diseases, having previous abstinence, living with family, cohabitation with smokers, desire to smoke at the start of treatment, self-efficacy at the start of treatment, desire to smoke at 4 weeks, self-efficacy at 4 weeks showed significant odds ratio for success of smoking cessation, although there was no statistically significant association (p>0.05). Similarly, male, age at smoking initiation, cigarettes smoked per day, having previous abstinence, living with family, cohabitation with smokers, desire to smoke at the start of treatment, self-efficacy at the start of treatment, desire to smoke at 12 weeks, self-efficacy at 12 weeks and CES-D score at 12 weeks showed significant odds ratio for smoking relapse, although there was no statistically significant association (p>0.05). In addition, our study suggests that varenicline is more effective than nicotine patch in the SCT program. Further research is expected to clarify the effect of providing financial support and the factors associated with smoking cessation and relapse in the SCT program based on financial support.
